# Design and Electromagnetic Properties of a Conformal Ultra Wideband Antenna Integrated in Three-Dimensional Woven Fabrics

**DOI:** 10.3390/polym10080861

**Published:** 2018-08-03

**Authors:** Ye Kuang, Lan Yao, Sheng-Hai Yu, Shuo Tan, Xiu-Jun Fan, Yi-Ping Qiu

**Affiliations:** 1 Key Laboratory of Textile Science & Technology (Donghua University), Ministry of Education, Shanghai 201620, China; 1135004@mail.dhu.edu.cn; 2 College of Textiles, Donghua University, Shanghai 201620, China; yush1106@163.com (S.-H.Y.); tanshuo123@126.com (S.T.); 18301957959@163.com (X.-J.F.); ypqiu@dhu.edu.cn (Y.-P.Q.)

**Keywords:** textile antenna, ultra wideband antenna, three-dimensional woven fabric, smart textiles, electromagnetic properties

## Abstract

Wearable antennas play an important role in transmitting signals wirelessly in body-worn systems, helping body-worn applications to achieve real-time monitoring and improving the working efficiency as well as the life quality of the users. Over conventional antenna types, ultra wideband (UWB) antennas have advantages of very large operating bandwidth, low power consumption, and high data transmission speed, therefore, they become of great interest for body-worn applications. One of the strategies for making the antenna comfortable to wear is replacing the conventional rigid printed circuit board with textile materials in the manufacturing process. In this study, a novel three-dimensional woven fabric integrated UWB antenna was proposed and fabricated with pure textile materials. The antenna electromagnetic properties were simulated and measured and its properties under bending were investigated. The antenna operated in a wide bandwidth from 2.7 to 13 GHz with the proper radiation pattern and gain value. At the same time, the antenna performance under bending varied in a reasonable range indicating that the antenna is prospectively applied on the curved surfaces of the human body. Additionally, the current distribution of the antenna showed that different conductive parts had different current densities indicating the uniqueness of the three-dimensional textile-based antenna.

## 1. Introduction

Body-worn applications are enhancing the working efficiency and improving the daily life quality of people by real-time monitoring the motion, movement, physiological indices, and the surrounding environment of the users [1,2,3,4,5]. To acquire the data captured from wearable applications continuously and remotely with minimum effects on the user, an antenna module either miniature in size or flexible is preferred to be used to transmit the data wirelessly [6,7]. In order to achieve the comfort requirements of a wearable antenna, textile materials are thought to be promising alternatives of the conventional solid circuit board because of their flexible, lightweight, low-cost and ease of being unobtrusively integrated into garments [8,9,10,11]. Moreover, some special wearable antennas are required to have good impact tolerance and durability especially in harsh environments, such as fire scenes and battlefields. As a high-performance textile material having good wearability and extraordinary strength, aramid fiber is much stronger than common textile materials and generally used in body armor, which is also preferred in the wearable antenna substrate. Integrating the antenna into three-dimensional woven fabric makes a seamless and robust textile antenna structure possible. The concept of a three-dimensional woven antenna was proposed in 2015, and in that structure the conductive and substrate layers were bound tightly together, which gave the antenna a low risk of delamination and increased the resistibility to mechanical damage, thus improving the durability of the textile antenna [12].

An UWB antenna refers to an antenna that has −10 dB bandwidth of at least 500 MHz, or 0.2 in fraction form [13]. Since the Federal Communications Commission (FCC) released the unlicensed frequency band of 7.5 GHz from 3.1 to 10.6 GHz for UWB communication in 2002, UWB antennas have attracted much attention from industry and academia. The UWB antenna has several unique advantages, such as very large operating bandwidth, low power consumption, and high data transmission speed. That makes UWB antennas a desirable choice for body-worn applications, especially for emergency use [14,15].

In this study, a conformal ultra wideband antenna integrated in three-dimensional woven fabrics was designed and fabricated. The conducting parts and the insulating substrate of the antenna were made up of copper yarns and aramid yarns respectively. The essential electromagnetic properties of the antenna, including the return loss, radiation pattern and gain were simulated and measured. Furthermore, the effects of bending on the antenna radiation properties were investigated considering the antenna would be applied on curved surfaces in certain body-worn applications.

## 2. Experimental

### 2.1. Antenna Design and Fabrication

**Figure 1:**
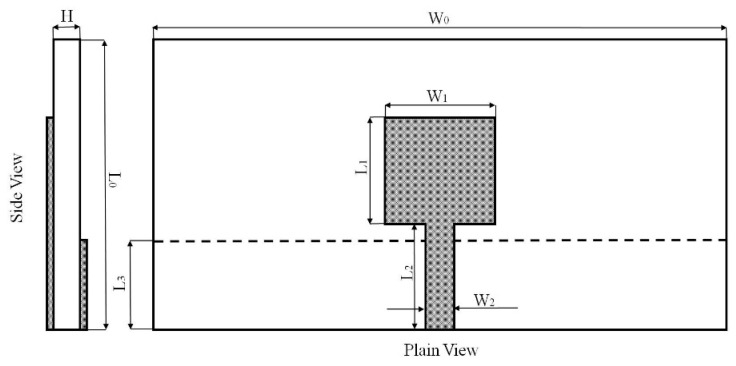
The configuration of the proposed antenna.

The antenna proposed in this study was designed covering the FCC UWB band from 3.1 to 10.6 GHz with the return loss higher than 10 dB. The configuration of the proposed antenna is illustrated in Figure 1. A planar rectangular radiation patch (*L*_1_ × *W*_1_) was constructed with a 50 Ω microstrip line (*L*_2_ × *W*_2_) in the front of the antenna, and a ground plane (*L*_3_ × *W*_0_) was partially covered at the back of the microstrip line. The substrate (*L*_0_ × *W*_0_ × *H*) was made up of aramid yarns in the middle of the antenna with dielectric constant and loss tangent of 1.96 and 0.042 respectively. The antenna was able to excite several overlapped resonant modes then broaden the antenna bandwidth, and the antenna tended to behave like a quarter-wavelength monopole antenna at the first resonant mode [16]. Therefore, the length of the rectangular radiation patch (*L*_1_) was possible to be estimated after the first resonant frequency was set according to the following equation:

(1) $$L_{1}=\frac{c}{4f\sqrt{\varepsilon_{\mathrm{reff}}}}$$

where *c* is the speed of light in free space, *f* is the resonant frequency in the first resonant mode and ε_reff_ is the effective dielectric constant of the substrate. The ε_reff_ is obtained by using the substrate relative dielectric constant ε_r_ as follow:

(2) $$\varepsilon_{\mathrm{reff}}\approx \frac{\varepsilon_{r}+1}{2}$$

After the patch length was estimated, a simulation model was established in simulation software ANSYS HFSS and the antenna size parameters were optimized and listed in Table 1.

**Figure 2:**
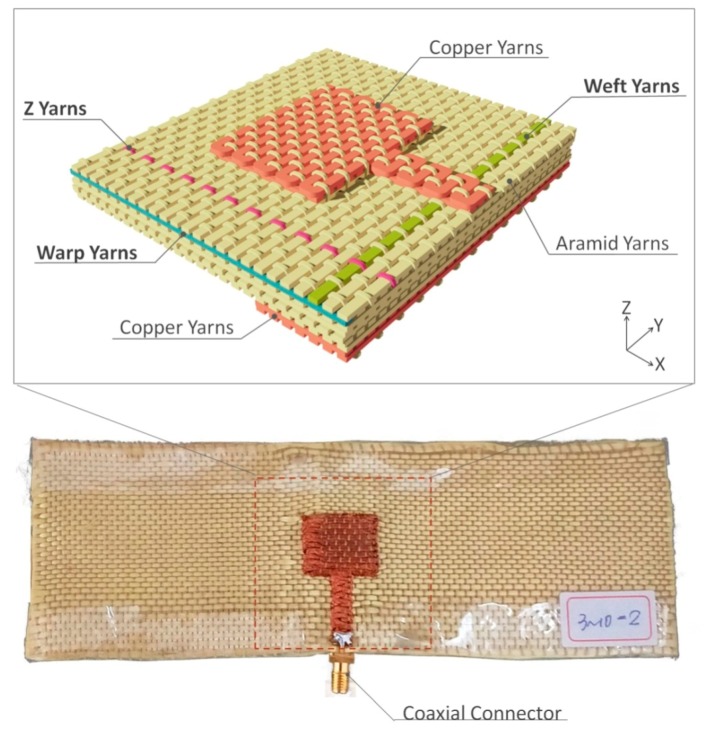
The photograph and woven structure of the proposed antenna.

The proposed antenna was fully integrated into a three-dimensional orthogonal woven fabric using three-dimensional weaving machine. The woven structure consisted of three groups of yarns which were warp, weft and Z yarns as shown in Figure 2. The radiation patch and microstrip line were fabricated together by using copper yarns on top of the structure. The partially covered ground plane consisted of copper yarns at the bottom of the structure. The antenna substrate was composed of three layers of warp yarns and four layers of weft yarns in the middle of the antenna structure. The entire antenna structure was bounded tightly together with a group of Z yarns which went through *Z* axis. The special yarn arrangement made the structure not only flexible but also having a low risk of delamination. The details of the yarn specifications and weaving parameters are listed in Table 2 [17]. After the textile structure was fabricated, a coaxial connector was soldered at the edge of the microstrip line for signal feeding.

### 2.2. Simulation and Measurment of Antenna Electromagnetic Properties

In this study, essential antenna electromagnetic properties including return loss, radiation pattern and gain were investigated. Return loss (*RL*) reflects the antenna impedance matching of which the expression in dB is:

(3) RL=−20log|Γ|

where Γ is the reflection coefficient. The return loss was measured by Agilent N5230A Microwave Network Analyzer (Agilent Technologies, Santa Clara, CA, USA) as *S*_11_ parameter, which was the reflection coefficient in dB at port 1 of the network analyzer. The radiation pattern of an antenna is a graph of radiation wave strength characterizing the far-field radiation performance at a specific frequency. Gain describes the ability of the antenna converting the input power into radio wave headed in a specific direction. For measuring the antenna radiation pattern and gain, the antenna was fixed with a termination of the section port in an anechoic chamber, and then the signal of the vertical polarization was transmitted from a measurement antenna to the textile antenna. The radiation pattern was obtained from the signal received by the 360° rotating antenna, and the gain was calculated by comparing field values of a reference-gain horn antenna after the radiation pattern was obtained.

The simulation of the antenna electromagnetic properties was used for optimizing the antenna size parameters as well as estimating the antenna performance before the antenna was fabricated. The software used for antenna electromagnetic properties simulation in this study was ANSYS HFSS. In addition to the essential antenna electromagnetic properties mentioned previously, antenna current distribution was also simulated for investigating the influence of the woven structure on antenna radiation.

## 3. Results and Discussion

### 3.1. Return Loss

**Figure 3:**
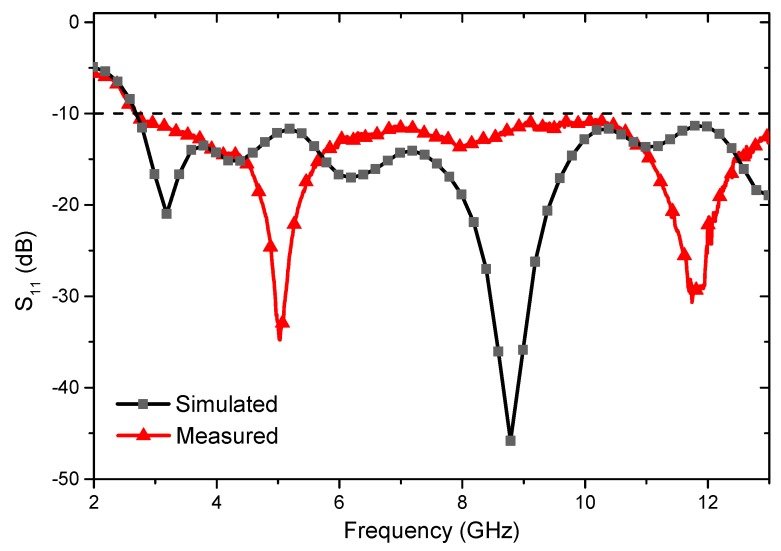
Simulated and measured return loss graphs of the proposed antenna.

As plotted in Figure 3, the antenna had a wide simulated −10 dB bandwidth from 2.7 to 13 GHz and two simulated resonant frequency points at 3.2 and 8.9 GHz. The measured −10 dB bandwidth was similar to the simulated results, however, the two measured resonant frequencies went up to 5.1 and 11.7 GHz, respectively. The increase of the measured resonant frequencies may result from the inaccurate dimension control of the radiation patch and microstrip line and the millimeter-sized manufacturing error showed significant influence in antenna properties. The inaccurate dimension control could be improved by using size controlling equipment in the antenna fabrication process. The special woven structure was another reason of resonant frequency shifting because the current was found to be concentrating on the inner thin warp yarns for the three-dimensional woven microstrip structure, which led to the current path changing [17].

### 3.2. Radiation Pattern and Gain

**Figure 4:**
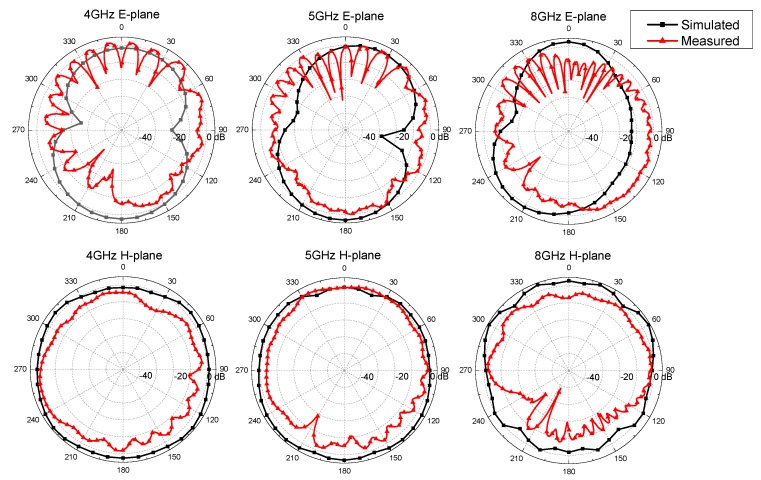
The simulated and measured radiation patterns of the proposed antenna.

The far-field properties of the proposed antenna were investigated in its first resonant point of 5 GHz and representative low and high frequency points of 4 and 8 GHz. Simulated and measured E-planes (XOZ planes) and H-planes (YOZ planes) of the antenna radiation patterns are plotted in Figure 4. For the simulated results in 4 and 5 GHz, the antenna had the highest radiation power at around 0 and 180 degrees in E-planes and approximate omnidirectional behavior in the H-planes. The measured E-planes at 4 and 5 GHz had more ripples around 0 degrees and stronger radiation strength around 90 and 270 degrees than the simulated results. The measured H-planes at 4 and 5 GHz having approximate omnidirectional radiations were similar to the corresponding simulated results, although the smaller distortions were observed in the simulated results. When the frequency was increased to 8 GHz, larger radiation pattern distortions appeared in both simulated and measured results. Overall, the measured H-planes showed better agreement with the simulated results than E-planes and the antenna had more uniform radiation at 4 and 5 GHz than at 8 GHz.

The simulated and measured gains of the proposed antenna at 4, 5, and 8 GHz are listed in Table 3. The difference between the measured and simulated results may be explained by the radiation pattern distortion and radiation strength decline. At 4 and 5 GHz, the measured gains had higher values than simulated results because the radiation showed higher concentrations in certain angles and made the radiation strength stronger. The measured gain at 8 GHz was lower than the simulated results because the high dielectric loss of the aramid substrate and high conductivity loss of the woven radiation patch in high frequency showed major effects on antenna radiation. The discrepancies between measured and simulated antenna gains were 3.44, 1.16, and 3.67 dBi at 4, 5 and 8 GHz, which were reasonable values when compared with other three-dimensional woven antennas proposed in [12,17].

### 3.3. Current Distribution

**Figure 5:**
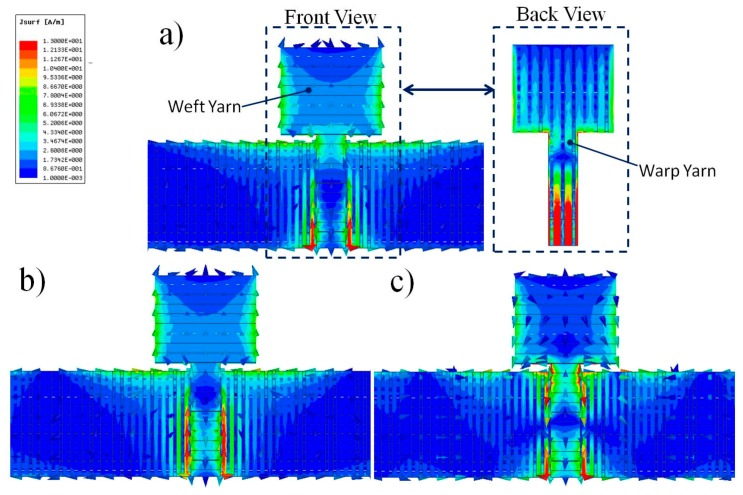
Simulated antenna current distribution: (**a**) 4 GHz; (**b**) 5 GHz; and (**c**) 8 GHz. The values correspond to the case when the antenna accepts 1 W of power.

Figure 5 illustrates the simulated current distributions of the antenna at 4, 5, and 8 GHz. As shown in Figure 5a, the current had different distributions at the front and back surfaces of the conductive layer because the three-dimensional woven antenna consisted of two groups of orthogonally-placed yarns. For the microstrip line used for signal transmission, the current mainly concentrated on the surface of inner warp yarns. For the radiation patch without the ground plane covered at the back of the substrate, the current densities were similar on the surface of inner warp yarns and outer weft yarns. However, the gaps between the warp yarns were larger than the gaps between weft yarns in the three-dimensional woven structure. Therefore, the inner layer and outer layer of the radiation patch contributed differently to antenna radiation and then resulted in variations in the electromagnetic properties.

The current distribution of the antenna varied under different frequencies as illustrated in Figure 5a–c. In 4 and 5 GHz, the current mainly concentrated on the right and left edges of antenna radiation patch, outer edges of microstrip line and upper edge of ground plane. When the frequency was increased to 8 GHz, the current densities of radiation patch and ground plane decreased and the current density of microstrip line increased with most of the current concentrating on the edges. The current distribution showed that the radiation of the antenna relied on the radiation excited from the edges of the conductive patterns. In order to obtain good antenna performance, the conductive pattern dimensions should be well controlled in the fabrication process. 

### 3.4. Bending Test

**Figure 6:**
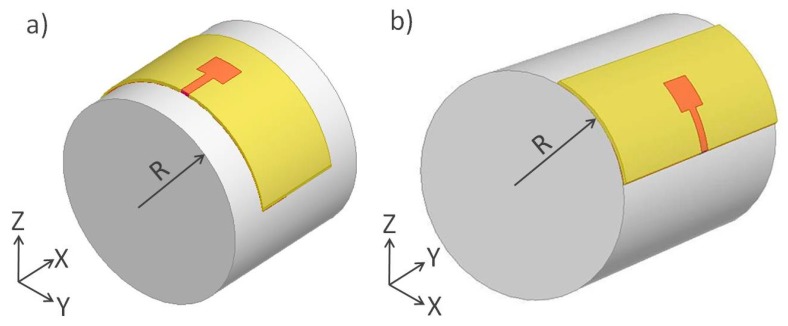
Schematic of the bending test along (**a**) the *X*-axis and (**b**) the *Y*-axis.

The effects of curvature on antenna performances were investigated by mounting the antenna on the surface of foam cylinders with different radii. There were three bending focus conditions of which the radiuses (*R*) were 5, 15, and 25 cm. Both *X*-axis bending and *Y*-axis bendings were investigated as illustrated in Figure 6.

**Figure 7:**
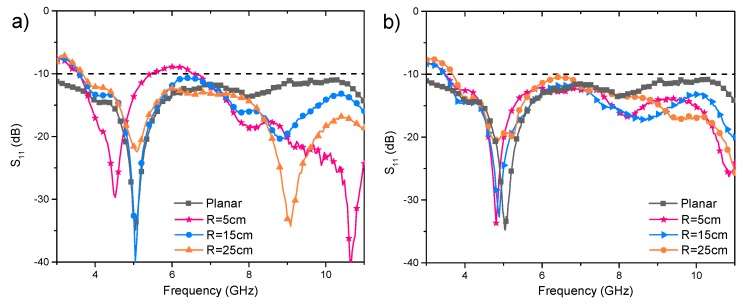
The return loss graphs of the proposed antenna under bending along (**a**) the *X*-axis and (**b**) the *Y*-axis.

Figure 7 shows the effects of bending along both the *X*-axis and the *Y*-axis on antenna return loss. When the antenna was bent along *X*-axis on the 25 and 15 cm foam cylinder, its first resonant frequency stayed at 5.1 GHz, but the second resonant frequency varied to around 9 GHz. When the 5 cm foam cylinder was used, the first and second resonant frequency decreased apparently and the return loss from 5.5 to 6.6 GHz became lower than 10 dB making the bandwidth narrower. In the *Y*-axis bending condition, the first resonant frequency showed a slight decrease with the cylinder radius reduced from 25 to 5 cm, the return loss between the first and second resonant frequencies became slightly higher after *Y*-axis bending. Overall, the curvature showed less effect on return loss in antenna curved along the *Y*-axis than that in antenna curved along *X*-axis, and both the *X*-axis and *Y*-axis bending showed slight effects on the antenna bandwidth. 

**Figure 8:**
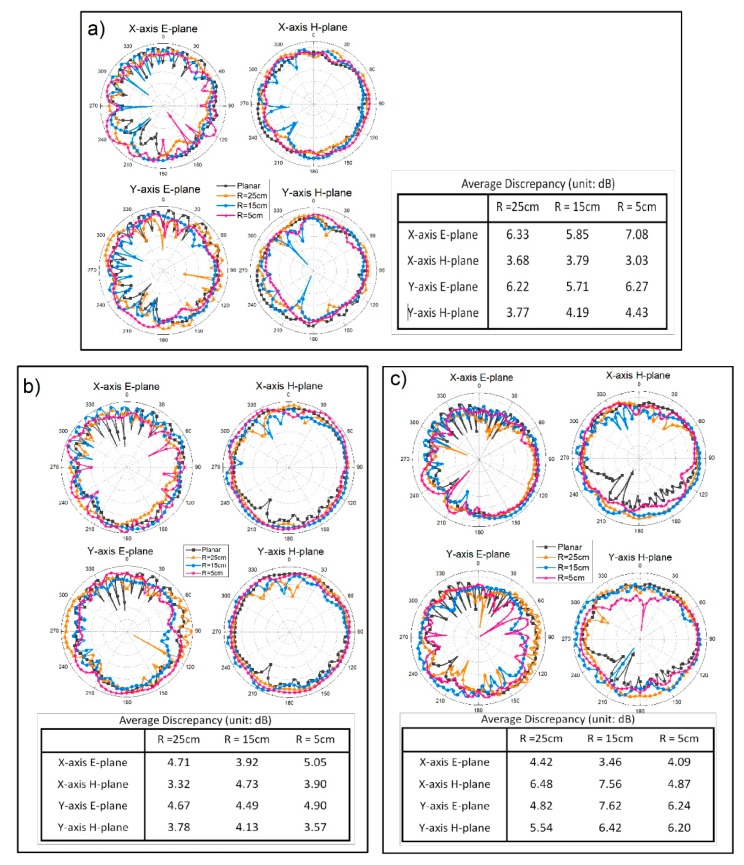
The radiation patterns of the proposed antenna under bending along the *X*-axis and the *Y*-axis, and the corresponding average discrepancies at (**a**) 4 GHz; (**b**) 5 GHz; and (**c**) 8 GHz.

Figure 8 shows the effects of bending along both *X*-axis and *Y*-axis on antenna radiation pattern. To present the discrepancies between the radiation patterns of the curved antenna and planar antenna clearly, the average discrepancy ($\bar{D}$) was calculated as follows:

(4) $$\bar{D}=\frac{1}{n}\overset{n}{\underset{i=1}{\sum }}\left|A_{C,i}-A_{P,i}\right|$$

where *n* was the number of angels in measurement, *A*_C,i_ and *A*_P,i_ were the measured radiation amplitude of the curved antenna and planar antenna in angle *i*. According to the calculated results, *X*-axis bending and *Y*-axis bending showed similar effects on antenna radiation patterns, with smaller average discrepancies at 5 GHz and larger average discrepancies at 8 GHz. The antenna offered approximate omnidirectional radiation behavior after being curved along both the *X*-axis and *Y*-axis at 4, 5, and 8 GHz, and the maximum gain fluctuated over a limited range as listed in Table 4. 

## 4. Conclusions

In this study, a three-dimensional woven fabric integrated UWB antenna was designed and fabricated. The antenna had a wide −10 dB bandwidth from 2.7 to 13 GHz. The radiation pattern of the antenna was approximately omnidirectional and the gains of the antenna at 4, 6, and 8 GHz were 2.55, 1.38, and 0.15 dBi, which were appropriate for a three-dimensional woven fabric integrated antenna type. Bending tests were implemented on cylinders having radii from 5 to 25 cm along the *X* and *Y* axis. Although the *X*-axis bending showed a larger effect on antenna return loss than *Y*-axis bending, both of the bending conditions showed little influence on antenna bandwidth. In addition, the radiation patterns and gains after bending varied in a reasonable range indicating that the antenna had good resistivity against the influence of bending. As a new attempt at integrating an UWB antenna into a three-dimensional woven fabric, the differences between measured and simulated electromagnetic properties resulted from the inaccurate dimension control and special woven structure. The further improvement could be achieved by using a machine for antenna size control and optimizing the weaving parameters for a better woven structure.

## Figures and Tables

**Table 1:** The size parameters of the proposed antenna.

Parameters	*L*_0_	*W*_0_	*L*_1_	*W*_1_	*L*_2_	*W*_2_	*L*_3_	*H*
Size (mm)	56.2	160	15.8	18.3	20.4	5.3	19	1.7

**Table 2:** The specifications of the yarns and weaving parameters for the three-dimensional woven fabric integrated antenna.

Yarns	Yarns Density (ends per inch)	Yarns Thickness	
		Copper (mm)	Aramid (Kevlar 129) (dtex)
Warp yarns	18.4	0.3	1580
Weft yarns	15.4	0.8	1580
Z yarns	18.4	----	445

**Table 3:** The simulated and measured gains of the proposed antenna.

Frequencies (GHz)	Simulated Gain Values (dBi)	Measured Gain Values (dBi)	Discrepancies between Measured and Simulated Results (dBi)
4	−0.89	2.55	3.44
5	0.22	1.38	1.16
8	3.82	0.15	3.67

**Table 4:** The gains of the proposed antenna at 4, 5, and 8 GHz under bending along the *X*-axis and the *Y*-axis.

	Gains in 4 GHz		Gains in 5 GHz		Gains in 8 GHz	
	*X*-axis	*Y*-axis	*X*-axis	*Y*-axis	*X*-axis	*Y*-axis
Planar	2.55		1.38		0.15	
*R* = 25 cm	0.31	2.37	2.21	4.12	−0.70	3.80
*R* = 15 cm	1.60	−0.32	4.35	3.42	2.79	4.30
*R* = 5 cm	3.96	0.13	4.18	2.59	0.23	0.15
